# Circulating miRNAs as Putative Biomarkers of Exercise Adaptation in Endurance Horses

**DOI:** 10.3389/fphys.2018.00429

**Published:** 2018-04-24

**Authors:** Katia Cappelli, Stefano Capomaccio, Andrea Viglino, Maurizio Silvestrelli, Francesca Beccati, Livia Moscati, Elisabetta Chiaradia

**Affiliations:** ^1^Dipartimento di Medicina Veterinaria, Centro di Studio del Cavallo Sportivo, University of Perugia, Perugia, Italy; ^2^Facoltà di Scienze Agrarie, Alimentari e Ambientali, Istituto di Zootecnica, Università Cattolica del Sacro Cuore, Piacenza, Italy; ^3^Istituto Zooprofilattico Sperimentale dell'Umbria e delle Marche, Perugia, Italy

**Keywords:** miRNA, endurance, NGS, biomarkers, horses

## Abstract

Endurance exercise induces metabolic adaptations and has recently been reported associated with the modulation of a particular class of small noncoding RNAs, microRNAs, that act as post-transcriptional regulators of gene expression. Released into body fluids, they termed circulating miRNAs, and they have been recognized as more effective and accurate biomarkers than classical serum markers. This study examined serum profile of miRNAs through massive parallel sequencing in response to prolonged endurance exercise in samples obtained from four competitive Arabian horses before and 2 h after the end of competition. MicroRNA identification, differential gene expression (DGE) analysis and a protein-protein interaction (PPI) network showing significantly enriched pathways of target gene clusters, were assessed and explored. Our results show modulation of more than 100 miRNAs probably arising from tissues involved in exercise responses and indicating the modulation of correlated processes as muscle remodeling, immune and inflammatory responses. Circulating miRNA high-throughput sequencing is a promising approach for sports medicine for the discovery of putative biomarkers for predicting risks related to prolonged activity and monitoring metabolic adaptations.

## Introduction

Endurance exercise stimulates metabolic and structural adaptations that primarily involve the musculoskeletal, cardiovascular, respiratory, endocrine, and immune systems (Gleeson, 2006; Ellison et al., 2012; Kirby and McCarthy, 2013; Rowe et al., 2014; Booth, 2015; Black et al., 2016) and processes involved in muscle remodeling, mitochondrial synthesis and angiogenesis (Yan et al., 2011). Although these changes have been widely investigated, the cellular and molecular mechanisms mediating these adaptations are still not completely understood (Capomaccio et al., 2010a,b, 2011, 2013; Scoppetta et al., 2012; Cappelli et al., 2013; Mach et al., 2017).

Physical exercise has recently been associated with the modulation of a particular functional RNA class, micro RNAs (miRNAs), in human skeletal muscles (Diniz and Wang, 2016), mice liver (Ohde et al., 2016), human leucocytes (Radom-Aizik et al., 2013; Makarova et al., 2014), and human cardiomyocytes (Souza et al., 2015) and mice liver (Ohde et al., 2016).

MicroRNAs are small noncoding RNAs (18–22 nucleotides) that act as post-transcriptional regulators of gene expression, modulating numerous biological processes, including cellular proliferation, differentiation and metabolism (Aoi and Sakuma, 2014). Their genomic context spans introns of protein coding genes, introns and exons of noncoding RNA genes and occasionally exons of protein coding genes. Their binding sites are usually in the 3′UTR and rarely in the 5′UTR or coding region of mRNA sequences (Finnegan and Pasquinelli, 2013). Each miRNA can regulate the expression of hundreds of genes and proteins, while a single gene may be regulated by several miRNAs (Friedman et al., 2009). They can be also released into body fluid in stable forms—termed circulating miRNAs (ci-miRNAs)—that are associated with different RNA-binding proteins or lipoprotein complexes, are actively incorporated into microvesicles and apoptotic bodies and are passively released as the result of cell death (apoptosis or necrosis), autophagy and cell rupture (Xu et al., 2015; Ghai and Wang, 2016). Circulating miRNAs function in transcriptional gene silencing but also act like hormones, in signal transduction by binding Toll-like receptors, (Fabbri et al., 2013).

This specific and controlled cell discharge indicates that released miRNAs are involved in the regulation of pathophysiological mechanisms and that ci- miRNAs are an evolutionarily conserved tool for cell-cell communication, providing fast local or systemic responses to perturbations in homeostasis (Kosaka et al., 2013). On the basis of these findings, ci-miRNAs have been proposed as biomarkers for the diagnosis and prognosis of diseases, as well as for monitoring therapeutic treatments (Ghai and Wang, 2016).

Circulating-miRNAs in the bloodstream could be also used to evaluate the response to training protocols and as molecular predictors of exercise performance in human and equine athletes (Keller et al., 2011; Lombardi et al., 2016; Mach et al., 2016; Polakovičová et al., 2016) because ci-miRNAs plasma levels depend on the type and duration of physical exercise, on the training level and the athlete's nutrition (Kirby and McCarthy, 2013; Makarova et al., 2014; van der Kolk et al., 2015). They also provide greater accuracy than classical serum/plasma biomarkers such as the proteins creatine kinase or lactate dehydrogenase (Makarova et al., 2014) as they are easily affected by physical stress, while those proteins, although consistent markers of muscle metabolism, are partially involved in the cellular response to environmental stimuli (Baggish et al., 2014). In addition, ci-miRNAs are highly stable and are resistant to variations in temperature and pH and to multiple freeze/thaw cycles, making sample storage, and handling easy (Polakovičová et al., 2016).

Various miRNA profiling methods can be used (e.g., qRT-PCR, microarray, sequencing), and they differ in terms of robustness, throughput, accuracy, sensitivity, dynamic range, cost and complexity. The major advantages of miRNA-sequencing with next-generation sequencing (NGS) are the ability to detect both novel and known miRNAs, the ability to perform relative quantification of miRNA transcripts present at a low abundance and the fact that miRNA variant characterization can usually be performed in a single experiment, which is especially useful in early biomarker discovery efforts (Ghai and Wang, 2016; Lombardi et al., 2016). To date, this approach has not been applied to profiling ci-miRNA fluctuations in response to endurance exercise.

The aim of this study was to assess the serum profile of ci-miRNAs in response to prolonged endurance exercise in samples obtained from competitive horses through massive parallel sequencing. Tracking the extent of ci-miRNA modulation could improve our understanding of the molecular mechanisms involved in the whole-body adaptive response to endurance exercise and provide useful potential biomarkers to predict disease risks related to prolonged activity (i.e., poor performance), as well as track metabolic adaptations to ultimately establish efficient training programmes.

## Materials and methods

### Ethics statement

This study protocol has been reviewed and approved by institutional Ethics Committee of the University of Perugia (license No 2016-11). All procedures were performed in accordance with the approved guidelines. Informed consent from the owners and the approval of the Ground Jury President and the Veterinary Commission President of the event were obtained before initiation of any procedures involving the animals.

### Animals

Four trained pure-breed Arabian horses (3 females and one gelding reared in Sardinia, Italy) taking part in the same 90 km endurance competition were enrolled. Three horses were 6 years old, and one was 9 years old. All horses were stabled and trained in the same training stable and were subjected to the same management practices at the stable and throughout the ride.

### Training programmes and competition

The training activities before the competition included exercise with a horse walker every day flat work at lunge and/or in the arena, a training session of variable intensity every other day and a training session at the racetrack. Before the start of the endurance competition, the horses passed the compulsory examination performed according to the rules of the Italian Equestrian Federation (FISE). Animals were fed 1.5 h before the start of the competition with hay and concentrate pellets. In accordance with the FEI and FISE rules, all horses underwent veterinary inspections during the competition every 20–40 km, followed by 40-min periods during which the animals were provided with water, hay and a small amount of concentrate pellets. The mean recovery time (time from arrival at the entrance of the veterinary gate for intermediate veterinary inspections) was 125 s and ranged from 115 to 145 s. All horses successfully completed the race without complication; the average race speed was 17.221 km/h (range: 15.777–21.361 km/h).

### Plasma samples

Peripheral blood samples were obtained by jugular venipuncture at rest just before exercise (T0) within 2 h after the end of an acute exercise bout (T1). Blood was collected in vacutainers containing EDTA (two tube) or a serum clot activator (one tube). One EDTA tube was used to measure haematocrit (HCT), hemoglobin (HGB) concentration, red blood cell (RBC) count, and white blood cell (WBC) count by using the hematology analyser (EosBIO, Italy). The second one EDTA tube was centrifuged at 3,000 × *g* for 15 min to obtain plasma, which was then stored at −80°C until ci-miRNA analysis. Blood without anticoagulant was left to coagulate and was then centrifuged (10 min at 3,000 × g); the serum obtained was stored at −80°C until being used for creatine phosphokinase (CPK), lactate dehydrogenase (LDH), HGB, and total protein measurements. These tests were assessed by a Konelab 2001 (Sclavo, Italy) biochemical analyser using specific kits (Sentinel Diagnostics, Milan, Italy) according to the manufacturer's instructions.

### RNA isolation from plasma

Plasma (500 μl) was centrifuged with 3,000 × g for 5 min in order to pellet debris and cells. Then, RNA was isolated from the supernatant using the miRCURY ™ RNA Isolation Kit—Biofluids (Exiqon, Vedbaek, Denmark), and 1 μl of Spike-in mix (UniSp2, UniSp4, UniSp5 RNA) (Exiqon, Vedbaek, Denmark) was added to monitor RNA isolation.

### Library preparation and next-generation sequencing

A total of 6 μl of extracted RNA was converted into microRNA NGS libraries using a NEBNEXT library generation kit (New England Biolabs Inc.) according to the manufacturer's instructions. Each individual RNA sample had adaptors ligated to its 3′ and 5′ ends and was reversely transcribed into cDNA. Then, the cDNA was pre-amplified with specific primers containing sample-specific indexes. After an 18-cycle pre-PCR, the libraries were purified on QiaQuick columns, and the insert efficiency was evaluated using a Bioanalyzer 2100 instrument on high-sensitivity DNA chips (Agilent Inc.,). The microRNA cDNA libraries were size fractionated on a LabChip XT (Caliper Inc.,), and bands representing adaptors and 15–40 bp inserts were excised following the manufacturer's instructions. Samples were then quantified using qPCR and concentration standards. Based on the quality of the inserts and the concentration measurements, the libraries were pooled in equimolar concentrations (all concentrations of libraries to be pooled were the same). Finally, the library pools were quantified again using qPCR, and optimal concentrations of the library pools were used to generate clusters on the surface of a flow cell before sequencing using the v3 sequencing methodology according to the manufacturer's instructions (Illumina Inc.,). Samples were sequenced with an Illumina NextSeq 500 system, producing 50-nucleotide single-end reads.

### Raw data bioinformatic analysis

Following sequencing, we performed a data quality control step using FastQC (http://www.bioinformatics.babraham.ac.uk/projects/fastqc).

**Figure 1:**
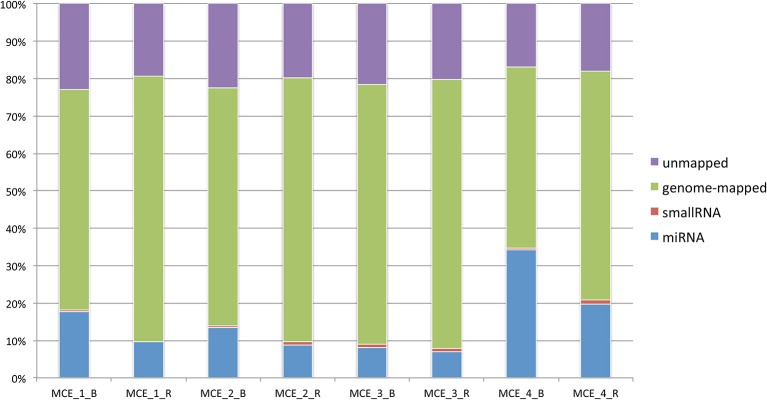
Classification of reads per sample based on the genomic compartment and proportion of mapped/unmapped sequences.

Mapping against equcab2 (*Equus caballus* assembly ver. 2) was carried out using Bowtie2 ver 2.2.5 (Langmead and Salzberg, 2012) in a multistep process to remove spike-in reads. The classification of reads is depicted in Figure 1 (data available in **Table 2**). Annotation of mapped regions was performed using reference annotations (miRBase 21).

After mapping the data and counting the relevant entries in miRBase 21, the number of known microRNAs was calculated. Comparisons of two groups considered all the data collected.

### Differential gene expression analyses

The differential gene expression (DGE) analysis was carried out using the edgeR statistical software package (Robinson et al., 2010) comparing the two groups applying a GLM procedure that can be seen as a generalization of a paired *t*-test. Normalization was carried out using the trimmed mean of *M*-values method based on log2-fold changes and absolute gene-wise changes in expression levels between samples (TMM normalization). Expression levels were measured as tags per million (“TPM”), and *p*-values for significantly differentially expressed microRNAs were estimated using an exact test on the negative binomial distribution.

### Target genes and enrichment analysis

For target analyses, we selected 12 miRNAs, the 6 most down-regulated and the 6 most up-regulated miRNAs according to the DGE analysis from edgeR. Human orthologue miRNAs were retrieved using the miRBase software association exchanging tool, and non-matching entries were classified using the BLAST algorithm and microRNAviewer software (http://people.csail.mit.edu/akiezun/microRNAviewer/index.html).

Putative genes (predicted and/or validated) targeted by these 12 miRNAs were identified using human miRNA IDs in the miRWalk webtool (Dweep and Gretz, 2015). Only genes included in the seven available databases (miRWalk, PITA, MicroT4, RNA22, miRanda, RNAhybrid and TargetScan) were retained in the analysis. For miRNAs without gene targets, we used a validated list of genes downloaded from the latest database update (DIANA Tarbase V. 7.0, Vlachos et al., 2015). Validated gene targets were also downloaded from the miRWalk 2.0 website. A unique list that merged both predicted and validated targets was produced for each miRNA, and common genes between different lists were identified.

The Cytoscape suite (Shannon et al., 2003) was used to build a protein-protein interaction (PPI) network and the PSIQUIC service (http://www.ebi.ac.uk/Tools/webservices/psicquic/view/home.xhtml) was used to download proteins interactions from six different public databases (ChEMBL, Spike, GeneMania, mentha, MINT, IntAct). These six networks were merged together in order to generate a unique PPI network with all the interactions. Only *Homo sapiens* interactions were used to build the new network, to avoid loss of information using equine data only.

The application clusterMaker 2.0 (Morris et al., 2011) with the “gLay” option was used to find different clusters inside the network according to the number of connections between nodes. Clusters were explored for their GO biological process enrichment terms using the application BiNGO, filtering for results with a corrected *q*-value lower than 0.05 (Benjamini Hochberg correction).

**Figure 2:**
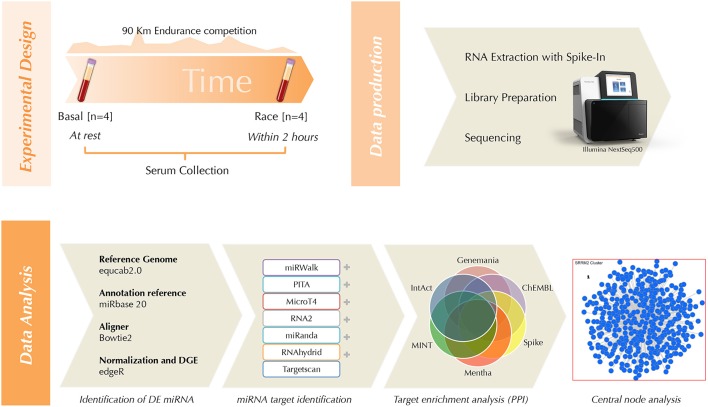
Workflow cartoon.

An overview of the experimental design and applied methods is depicted in Figure 2.

## Results

### Hematological and biochemical parameters

All horses included in the study completed a race and successfully passed the final veterinary examination at the veterinary gate. Changes in average hematological and biochemical parameters measured in blood samples taken before and after the race (Table 1) reflect the physical and metabolic effort that occurred during the endurance competition.

**Table 1:** Hematological and biochemical parameters evaluated from the two time points.

**Parameter**	**T0**	**T1**
Haematocrit (HCT) (%)	30.93 ± 4.38	34.55 ± 1.96
Red blood cell count (RBC) (1012/L)	7.61 ± 0.99	8.54 ± 0.4
WBC (109/L)	7.45 ± 1.529	11.45 ± 1.74
Hemoglobin (HGB) (g/dL)	12.7 ± 1.58	14.28 ± 0.73
Total protein (g/L)	64.979 ± 0.78	68.56 ± 4.69
Creatine kinase (CK): (IU/L)	376.04 ± 164.63	1499 ± 584.3
Lactate dehydrogenase (LDH) (IU/L)	697.3 ± 114.7	973.5 ± 146.8
Creatinine (μmol/L)	105.1 ± 19	137.8 ± 7.30

*Values are expressed as the means ± standard deviation*.

### Sequencing results

The data were quality checked for intensity correction, base calling and the assigning of Q-scores: the samples showed an overall high quality, with the vast majority of the sequences obtained presenting a Q-score higher than Q30. Raw data are available through BioProject with the following accession ID: PRJNA434539.

In the spike-in quality control step we observed an excellent correlation between the samples for counts corresponding to the spike-in values (*R*^2^ > 0.99, see Supplementary Figure 1).

On average, 29 million reads were obtained per sample, and the lowest number of reads for any sample was 12.9 million. Summary statistics for reads and mapping are detailed in Table 2. After mapping the sequences and counting occurrences of relevant entries in miRBase 21, the number of known microRNAs was calculated (Table 3). Raw count data and TPM are available in Supplementary Table 6.

**Table 2:** Summary statistics of sequencing, mapping and abundance on genomic features.

**Sample**	**Raw**	**miRNA**	**SmallRNA**	**Genome-mapped**	**Unmapped**
MCE_1_B	19,078,874	3,161,703	94,334	10,535,834	4,079,381
MCE_1_R	14,596,928	1,301,294	99,310	9,610,485	2,606,493
MCE_2_B	123,496,039	15,888,016	511,968	74,930,350	26,518,838
MCE_2_R	18,843,309	1,554,267	158,941	12,657,393	3,523,468
MCE_3_B	13,812,695	1,021,702	131,614	8,860,419	2,743,450
MCE_3_R	19,837,801	1,292,045	192,447	13,402,945	3,782,183
MCE_4_B	12,974,783	4,121,602	49,005	5,818,753	2,032,964
MCE_4_R	13,421,050	2,468,816	133,191	7,659,988	2,256,235

**Table 3:** Quantities of RNAs listed per abundance, in number of counts and in transcripts per million kilobases (TPM).

**Grouping Quantity (all samples)**	**Quantity (all samples)**
RNAs with ≥10 counts on average per sample	176
RNAs with ≥50 counts on average per sample	121
RNAs in all samples ≥ 1 TPM	163
RNAs in all samples ≥ 10 TPM	100

### Differential gene expression analysis

After statistical analysis with edgeR, 102 entries (miRNA and smallRNA) were found to be differentially expressed at *T1* vs. *T0* at a significance of *q* < 0.05 and an absolute log2-fold change (logFC) >1 or < -1. Using these filters, 27 RNAs were identified as being up-regulated (logFC >1), and 75 were identified as being down-regulated (Supplementary Table 1).

Table 4 shows the individual results for the 20 most differentially expressed microRNAs (10 up-regulated and 10 down-regulated).

**Table 4:** The 20 most significantly differentially expressed microRNA between T0 and T1.

**GeneName**	**logFC**	***P*-Value**	**FDR**
eca-miR-206	7.05	1.14E-25	3.76E-23
eca-miR-208b	6.02	1.84E-15	3.04E-13
eca-miR-133a	5.31	3.42E-15	3.76E-13
eca-miR-1	6.04	1.02E-14	8.42E-13
eca-miR-133b	4.75	1.34E-11	8.82E-10
eca-miR-499-5p	3.40	2.13E-09	1.17E-07
eca-miR-95	3.44	4.33E-06	2.04E-04
eca-miR-224	3.11	6.05E-06	2.50E-04
eca-miR-361-3p	−2.12	6.26E-05	1.79E-03
eca-miR-1180	−2.17	6.47E-05	1.79E-03
eca-miR-486-3p	−2.27	6.51E-05	1.79E-03
eca-miR-504	−2.39	4.92E-05	1.79E-03
eca-miR-328	−2.42	8.34E-05	2.12E-03
eca-miR-100	2.08	9.76E-05	2.30E-03
eca-miR-296	−1.95	1.68E-04	3.26E-03
eca-miR-6529	−2.03	1.64E-04	3.26E-03
eca-miR-9177	−2.29	1.57E-04	3.26E-03
eca-miR-9021	−1.90	2.26E-04	3.93E-03
eca-miR-486-5p	−2.10	2.20E-04	3.93E-03
eca-miR-381	1.89	2.68E-04	4.21E-03

*Modulation is expressed in log2-fold change (logFC). Entries are sorted by FDR value*.

### Target genes and enrichment analysis

A subset of 12 miRNAs was created by choosing the 6 most up-regulated and the 6 most down-regulated miRNAs based on the false discovery rate threshold to produce a list of unique target genes using both predicted and validated targets (Supplementary Table 2 for up-regulated and Supplementary Table 3 for down-regulated). Then, common target genes were identified: only those targeted by 4 or more miRNAs were selected (Table 5). Another filter based on the number of interactions was applied: only entries with more than five connections were further evaluated.

**Table 5:** List of selected genes targeted by four or more up- or down-regulated miRNAs suitable for GO enrichment analysis.

**Target Gene**	**miRNAs**	
**UP-REGULATED**		
*BCL2L2*	miR-1, miR-133a-3p, miR-133b, miR-206	
*EGFR*	miR-1, miR-133a-3p, miR-133b, miR-206	
*LASP1*	miR-1, miR-133a-3p, miR-133b, miR-206	
*ONECUT2*	miR-1, miR-133a-3p, miR-133b, miR-206	
*PTMA*	miR-1, miR-133a, miR-133b, miR-206, miR-499b-3p	
*PURB*	miR-1, miR-133a-3p, miR-133b, miR-206	
*SEC62*	miR-1, miR-133a-3p, miR-206, miR-208b-3p	
*SGCD*	miR-1, miR-133a-3p, miR-133b, miR-206	
*SH3PXD2B*	miR-1, miR-133a-3p, miR-133b, miR-206	
*STX6*	miR-1, miR-133a-3p, miR-133b, miR-206	
*TAGLN2*	miR-1, miR-133a-3p, miR-133b, miR-206	
*ZNF280C*	miR-1, miR-133a-3p, miR-133b, miR-206	
**DOWN-REGULATED**		
*ANKRD52*	miR-331-3p, miR-361-3p, miR-486-3p, miR-486-5p, miR-504-5p	
*DCAF7*	miR-328-3p, miR-331-3p, miR-486-3p, miR-504-5p	
*PLXNA4*	miR-328-3p, miR-361-3p, miR-486-5p, miR-504-5p	
*SPEN*	miR-1180-3p, miR-328-3p, miR-331-3p, miR-361-3p	
*SRRM2*	miR-1180-3p, miR-331-3p, miR-361-3p, miR-486-3p, miR-504-5p	

**Figure 3:**
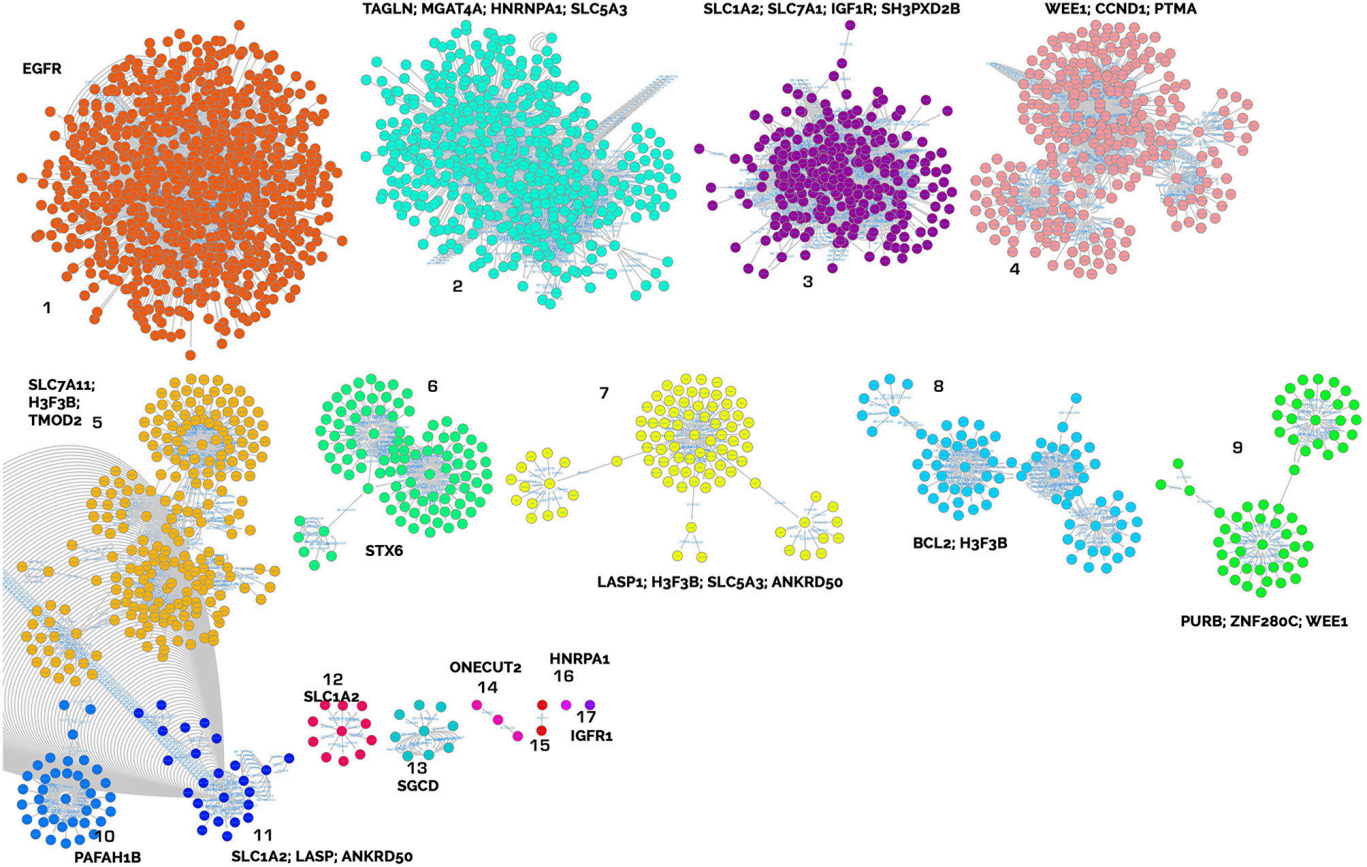
Clusters of proteins from the target analysis of up-regulated miRNAs. Each cluster is named with the central node protein name, targeted from the highest number of miRNAs.

**Figure 4:**
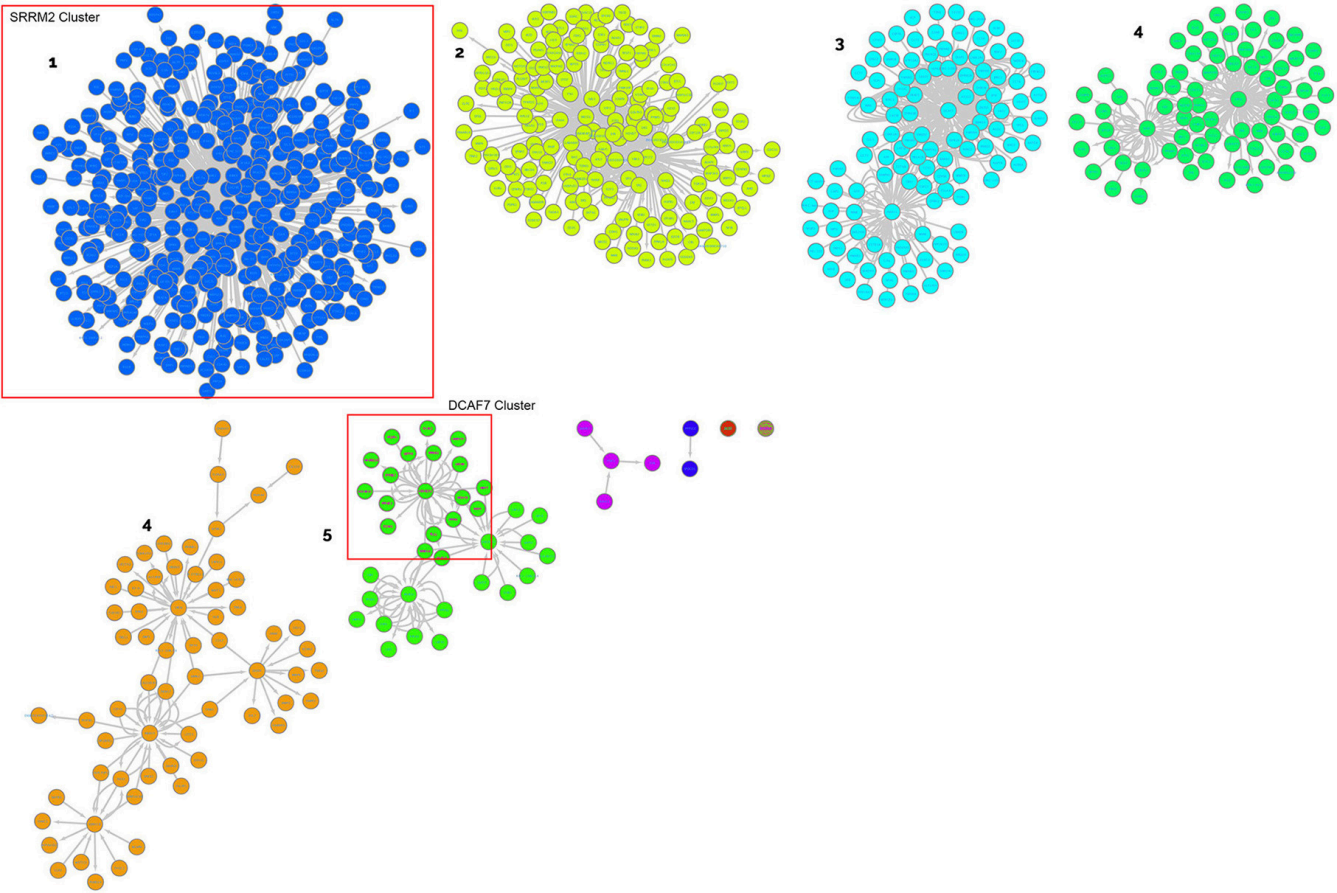
Clusters of proteins from the target analysis of down-regulated miRNAs. Each cluster is named with the central node protein name, targeted from the highest number of miRNAs.

These genes were used to create a PPI network as explained in the materials and methods section to identify clusters of proteins within the six merged network (merged in order to generate a unique PPI network with all the interactions) considering connections between nodes as selection criteria. This was done for both up- and down-regulated miRNA-derived targets. The network built with up regulated genes contains 2621 nodes and 8600 edges, while the network built with down regulated genes contains 861 nodes and 1535 edges (Supplementary Table 5). The major protein clusters are reported in Figures 3, 4.

Gene Ontology (GO) enrichment analysis with BiNGO was performed on the clusters harboring one of the targets or where a central node was targeted by several miRNAs and recognized as relevant in our biological framework (Table 6, and Supplementary Table 4).

**Table 6:** List of selected clusters of genes targeted by four or more up- or down-regulated miRNAs suitable for GO enrichment analysis.

**Cluster**	**Central Nodes**
**UP-REGULATED**		
1	*EGFR*	
2	*TAGLN; MGAT4A; HNRNPA1; SLC5A3*	
3	*SLC1A2; SLC7A1; IGF1R; SH3PXD2B*	
4	*WEE1; CCND1; PTMA*	
5	*SLC7A11; H3F3B; TMOD2*	
6	*STX6*	
7	*LASP1; H3F3B; SLC5A3; ANKRD50*	
8	*BCL2; H3F3B*	
9	*PURB; ZNF280C; WEE1*	
10	*PAFAH1B1*	
11	*SLC1A2; LASP1; ANKRD50*	
12	*SLC1A2*	
13	*SGCD*	
14	*ONECUT2*	
16	*HNRNPA1*	
17	*IGFR1*	
**DOWN-REGULATED**		
1	*SRRM2*	
5	*DCAF7*	

The key analyzed clusters belong to several meaningful biological processes and are summarized as follows:

- Muscle remodeling: IGF1R (insulin-like growth factor 1 receptor), cluster 17; EGFR (epidermal growth factor receptor), cluster 17; PURB (purine rich element binding protein B), cluster 9; TAGLN (transgelin), cluster 2; TMOD2 (tropomodulin 2), LASP1 (LIM and SH3 protein 1), cluster 7; and SGCD (sarcoglycan delta) cluster 13.
- Energy metabolism and cellular homeostasis maintenance: various solute carriers, such as SLC5A3 (sodium/myo-inositol cotransporter), clusters 1 and 7; SLC1A2 (excitatory amino acid transporter 2), clusters 3, 11, and 12; SLC7A1 (high affinity cationic amino acid transporter 1), cluster 3; and STX6 (syntaxin-6) cluster 6.
- Inflammatory response and modulation of cell migration: IGF1R, cluster 17; EGFR, cluster 17; BCL2 (B cell leukemia/lymphoma 2), cluster 3; and PTMA (prothymosin alpha), cluster 4.

## Discussion

This study profiles the amounts and types of ci-miRNAs in horse athletes competing in endurance races at rest and within 2 h after the end of competition, when the majority of the significant changes in ci-miRNAs occur (Nielsen et al., 2014). We identified the modulation of a large set of miRNAs involved in the response to exercise and inferred related target genes clustered in PPI networks that are correlated with different tissues (e.g., skeletal muscle, heart, liver and blood) and processes, such as muscle remodeling, metabolic changes and the modulation of immune and inflammatory responses.

Major changes were observed in circulating levels of muscle-specific miRNAs—collectively called myomiRs—derived from muscles and the heart: eca-miR206, 133a, 133b, 208b, 499-5p, and 486-3p. The miRNAs miR-206, 133a, 133b, 208b, and 499-5p were strongly up-regulated, with a high fold change values (+4 to +7), whereas miR-486 was the most down-regulated. This is consistent with previous studies, in which similar changes in levels of some myomiRs were observed in endurance human athletes after competition (Mooren et al., 2014; Russell and Lamon, 2015).

The changes in ci-miRNAs occurring in our athletes seemed to be a result of active or selective secretion rather than muscle cell damage. This is supported by increases in ci-miR-1, ci-133a, and ci-206, which are likely correlated with performance parameters and are poorly associated with biochemical markers of cardiac and/or skeletal muscle damage (Mooren et al., 2014), and by the decrease in the classical myomiR ci-miR-486 (Aoi et al., 2013; Xu et al., 2015).

Both adaptations to exercise and muscle regeneration involve myomiRs (Diniz and Wang, 2016) which act as modulators of myogenesis, mitochondrial biogenesis, hypertrophy and energy metabolism. For example miR-1 acts in the development and differentiation of smooth and skeletal muscle tissue together with miR-206 and miR-133. Overexpression of miR-1 or miR-206 promotes myogenic differentiation and the repair of exercise-induced muscle injury (Wu, 2016) and, along with the up-regulation of myomiR-133a, regulates mitochondrial biogenesis via the IGF-1-Akt (serine/threonine kinase 1) pathway through the IGF1R. In support of this, IGF1R was identified as a central node of cluster 17 (CNC 17) in our network analyses (Table 6, Figure 3). The down-regulation of mir-504 confirmed the presence of mitochondrial adaptation, which is essential in skeletal muscles involved in aerobic exercise. Namely, miR-504 acts as a negative regulator of p53, that has been implicated in regulating substrate metabolism and in exercise-induced mitochondrial biogenesis in skeletal muscle (Hu et al., 2010; Bartlett et al., 2014). These findings highlight the essential role of the previously described miRNAs in exercise tolerance and the response to training.

Cardiac muscle adaptations could involve EGFR (CNC 1, Table 6, Figure 3), plays roles in cell growth and proliferation, cardiac development, cardiac responses (adaptation/remodeling) to physiological and pathological loads. EGFR also modulates cardiomyocyte stress tolerance through the MAP kinase and phosphatidylinositol 3 kinase (PI3K)/Akt pathways (Reichelt et al., 2017). MicroRNAs regulate cardiac responses by targeting key components of these pathways. In our experiment, the up-regulation of mir-1 and mir-133 indicates the inhibition of pathological cardiac hypertrophy via the suppression of target genes in the heart and neural crest (Wang et al., 2016). Moreover, these two miRNAs appear to be particularly responsive to aerobic exercise and positively associated with VO_2_max (Mooren et al., 2014) and are associated with an increase in whole blood content in endurance athletes and with superior cardiorespiratory fitness levels (Clauss et al., 2016; Denham and Prestes, 2016).

The occurrence of general anti-hypertrophic conditions in our athletes during endurance racing is also suggested by the decrease in miR-328 associated with atrial fibrillation and cardiac hypertrophy (Li et al., 2014). In addition, up-regulation of myomiRs, as shown in our experiment, leads to an anti-hypertrophic signal (Soci et al., 2016) throughout regulation of *PURB*. PURB (CNC 9, Table 6, Figure 3) acts on the myosin genes and is considered a repressor of slow muscle contractions (Soci et al., 2016) that acts by controlling vascular smooth muscle alpha-actin gene transcription and repressing myoblasts (Hariharan et al., 2014).

Additionally miR-199 and the miR-99/100 family, which we found to be up-regulated and which are not strictly considered to be myomiRs, play a role in the maintenance of cardiac homeostasis and in regulating apoptosis in cardiomyocytes through their target, IGF1R (CNC 17, Table 6, Figure 3; Chen et al., 2015; Li et al., 2016).

Possible adaptive changes in cardiac tissue are also suggested by the increases in miR-499 and miR-208b, both of which are expressed in skeletal and cardiac muscles (Soci et al., 2016). These miRNAs, together with miR-206, act in the gene reprogramming process, activating slow myofibres and repressing fast ones, resulting in greater muscular endurance capacity (Kirby and McCarthy, 2013)_._ Muscle tissue remodeling also includes an increase in sympathetic and parasympathetic innervation involving muscle and cardiac autonomic nerve remodeling along with electrophysiological changes in the heart through the overexpression of miR-206 (Jeng et al., 2009; Zhang et al., 2015).

Structural modifications in muscles also occur via the regulation of stress responses and thyroid hormone signaling pathways involved in the modulation of myosin heavy chain genes thorough the overexpression of miR-208a (Baldwin and Haddad, 2001; Grueter et al., 2012). However, target and pathway analyses suggest that morpho-functional modifications of smooth, cardiac and skeletal muscles involve not only contractile proteins but also cytoskeletal proteins. Indeed, TAGLN, TMOD, and LASP1 (CNC 7, Table 6, Figure 3) act in stabilizing the contractile proteins and in regulating the dynamic actin cytoskeleton. Transgelin, is down-regulated by the cellular transformation involved in major cytoskeletal rearrangements, and it is implicated in smooth and cardiac muscle cell differentiation (Doll et al., 2007; Navickas et al., 2016). Tropomoduline, working in conjunction with tropomyosins, regulates the distribution and length of thin filaments in the sarcomeres of cardiac and skeletal muscle cells (Colpan et al., 2013; Rao et al., 2014). Moreover TMOD down-regulation, which occurred in our athletes, may contribute to the optimization of sarcomere length to maximize muscle activation and generate greater active forces (Kolb et al., 2016). Possible modifications of the sarcolemma may be achieved by SGCD (CNC, 13 Table 6, Figure 3), a protein that forms sarcoglycan complexes. These complexes play a key role in the mechanical stabilization of the sarcolemma, and they are modified in response to muscle contractions (Tarakci and Berger, 2016).

These results clarify the mechanisms underlying the massive structural and metabolic changes that generally occur in endurance athletes, which include an increase in mitochondria, fiber-type shifts and changes in capillary density (Kirby and McCarthy, 2013). Aerobic exercise training up-regulates miRNA expression to promote myoblast differentiation and proliferation and enables the metabolic reprogramming of skeletal and cardiac muscle cells to promote adaptations for improved physical performance (Denham and Prestes, 2016).

These anatomic changes all result in an increased ability to sustain prolonged physical activity, which also requires a greater energy supply. The reduction in ci-miR-486, which is proposed as a biomarker of advantageous adaptations to prolonged exercise, could be related to its specific uptake from blood into muscle cells (Aoi et al., 2013; Xu et al., 2015). The consequently high levels of miR-486 in these cells could result in enhanced muscle glucose uptake since the main target of this miRNA is phosphatase and tensin homolog (PTEN), a negative modulator of insulin signaling. Moreover, miR-486 can influence muscle protein turnover, acting as a modulator of the transcription factor FOXO (Xu et al., 2012, 2015; Kirby and McCarthy, 2013). However, independent of their specific roles and targets, the up-regulation of myomiRs in skeletal muscles after endurance exercise may be related to the decrease in muscle protein synthesis described in the post-exercise period (Rennie and Tipton, 2000), which may help to maintain cellular energy levels. Moreover, the involvement of SLC7A1 (CNC 12, Table 6, Figure 3), a non-hepatic trasponder of arginine, lysine and ornithine, indicates that amino acid uptake could also be modulated following endurance exercise.

The modulation of energy metabolism is also indicated by the involvement of SLC5a3, STX6, and IGFR1 (CNC 6 and 17, Table 6, Figure 3). The presence of SLC5a3, has recently been observed in cardiomyocytes, where it seems to act as a glucose sensor (Van Steenbergen et al., 2017), and STX6 can modify glucose uptake modulating GLUT4 translocation (Foley and Klip, 2014). Likewise, the deregulation of IGFR1 has consequences for insulin signaling and influences muscle glucose uptake.

The involvement of other solute carriers, such as SLC1A2 and SLC7A11 (CNC 5, Table 6, Figure 3), which act as Na+/glutamate and cysteine/glutamate transporters, respectively, suggests that exercise could regulate glutamate transport across membranes, as has been proposed by other authors, possibly to alleviate the neurotoxicity of excessive glutamate release (Yang et al., 2012).

Collectively, our results show activation of immune and inflammatory responses via the modulation of miR-224 and miR-1180 through a key immune systems regulator, transcription factor κB (NF-κB) (Yang and Wang, 2016). Indeed, inflammatory stimuli, such as strenuous exercise, can induce activation of the innate immune system via miR-224 and the pentraxin 3 *(Ptx3)* gene, both mediators of inflammation; miR-224 is a transcriptional target of NF-κB, and the *Ptx3* promoter contains an NF-κB binding site for its regulation (Rudnicki et al., 2014). The up-regulation of miR-224 leads to activation of EGF (its receptor is CNC 1, Table 6, Figure 3), transforming growth factor alpha (TGF-a), IGF (its receptor is CNC 17 Table 6, Figure 3) and tumor necrosis factor α (TNFα), inducing immune cell proliferation and migration via inflammatory response signals (Scisciani et al., 2012).

The down-regulation of miR-1180 suggests a modulation of immune cell survival and proliferation by directly targeting key inhibitors of the NF-κB signaling pathway and the apoptotic protein Bcl-2 (CNC 8, Table 6, Figure 3) (Tan et al., 2016). Moreover, PTMA (CNC 4, Table 6, Figure 3) acts in the immune response as an extracellular signaling molecule via Toll-like receptor 4 (Mosoian, 2011), decreasing the apoptotic response and increasing cell survival through activation of the serine-threonine kinase Akt (Cannavo et al., 2013).

## Conclusions

The modulation of protein coding genes in response to physical activity has been widely investigated, but a new level of regulation mediated by noncoding RNAs has only recently emerged as a key player in post-transcriptional regulation that may potentially be involved in adaptations in response to exercise.

We identified a large set of modulated ci-miRNAs and target genes that clustered in PPI networks, revealing that responses to endurance exercise induce major changes in muscle remodeling, metabolic pathways and, ultimately, in the immune system and the inflammatory response mechanisms. Therefore, modulated ci-miRNAs in endurance athletes can be considered promising and reliable biomarkers of stress and/or training.

Moreover, high-throughput ci-miRNA sequencing is a promising approach for use in sports medicine. The discovery of putative biomarkers for use in predicting disease risks related to prolonged activity (i.e., poor performance) and monitoring metabolic adaptations to establish efficient training schemes could be transferred to athlete of all species, including humans.

## Author contributions

KC, EC, SC, FB, and MS: coordinated the project and designed the study; KC, EC, LM, FB, AV, and SC: performed the experiments, interpreted the data and wrote the manuscript; SC, AV, KC, and EC: analyzed the sequencing data and performed the functional analyses; FB, LM, and MS: provided samples and interpreted clinical-hematological data. All the authors critically revised drafted manuscript and have read and approved in the final form.

### Conflict of interest statement

The authors declare that the research was conducted in the absence of any commercial or financial relationships that could be construed as a potential conflict of interest.
