# Supplementary material for: Circulating miRNAs as Putative Biomarkers of Exercise Adaptation in Endurance Horses
Source: Front Physiol. 2018 Apr 24;9:429. doi: 10.3389/fphys.2018.00429 (PMC5928201; doi:10.3389/fphys.2018.00429)

Supplementary Figure 1: correlation between the samples for counts corresponding to the spike-in values

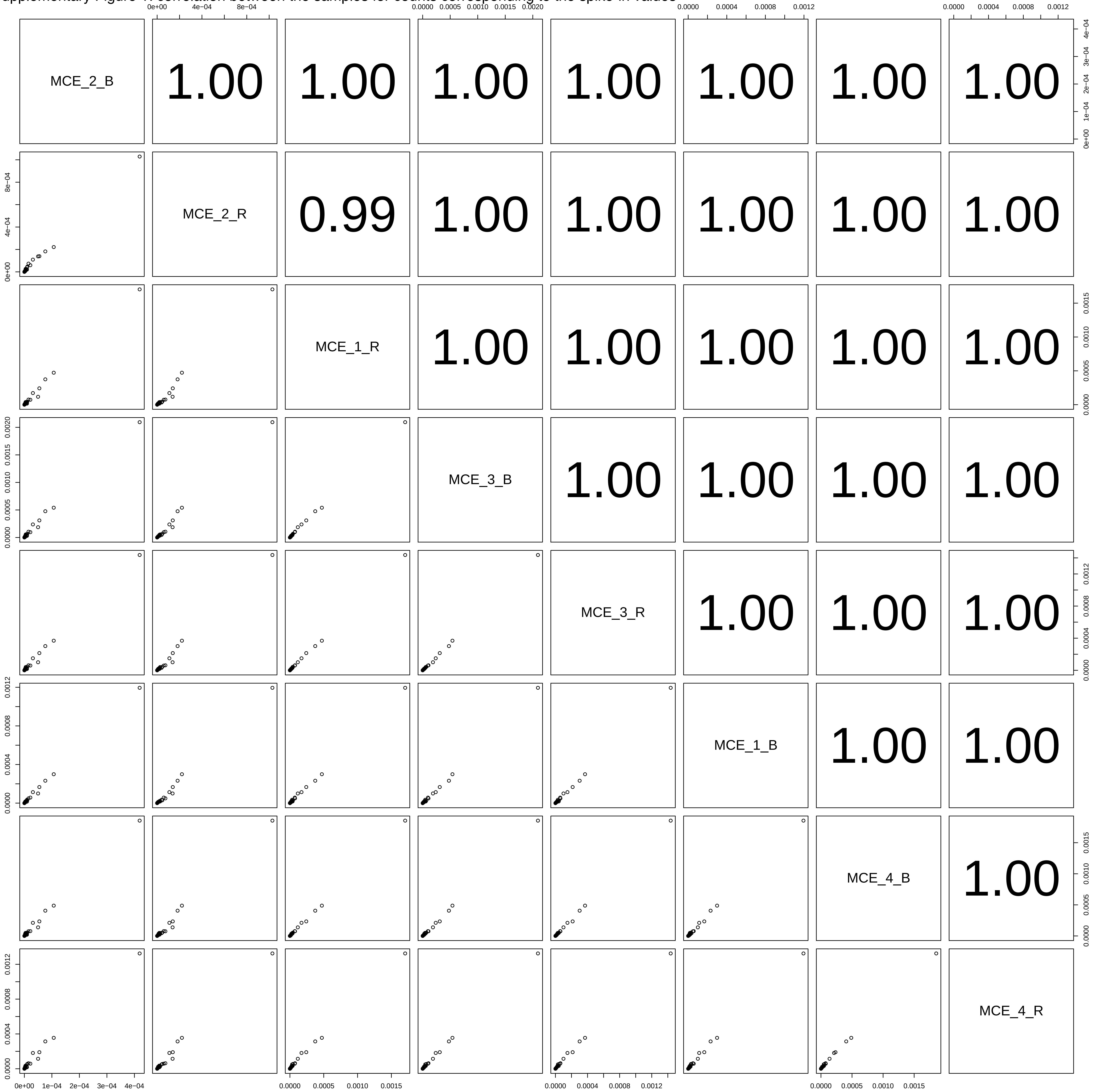

Supplement: Supplementary file 7 [file Image_1.pdf]
